# 

**Published:** 1882-06

**Authors:** 


### *Pennsylvania State Dental Society.*

The Fourteenth Annual Session of this society will be held July 25th, 26th, and 27th, at Williamsport, Pa. Arrangements have been made with the rail roads by which tickets can be secured at reduced rates, by presenting orders obtained from the Corresponding Secretary. The Park Hotel has reduced its figures from three to two dollars per day, to guest attending Convention.

Williamsport is delightfully situated on the North branch of the Susquehanna river, 70 miles North-west of Harrisburg, and is 900 feet above the sea; surrounded by some of Pennsylvania's finest mountain scenery, making it exceedingly picturesque, and with health giving atmosphere makes it an exceedingly pleasant resort.

The following programme has been prepared by the executive committee:

*Essays.*—President's address, C. B. Ansart, D. D. S., Oil City. "Lesions of the Alveolus," N. E. Magill, Erie. "The Influence of Constitutional Defects upon Dental Tissues," by C. S. Beck, M. D., D. D.S ., Wilksbare. "Nervous Force and Nervous Lesions" illustrated by vivisections, by N. C. Barrett, M. D., D. D. S., Buffalo. "What Constitutes Success in Dental Practice," by D. T. Nay, D. D. S., Bedford. "Reaction of Dentine with Special Relation to the Combination of Gold and Amalgam in Approximate Cavities," by H. C. Register, M. D. Philadelphia "Adjusting Artificial Crowns to Roots of Natural Teeth," by W. H. Fundenberg, D. D. S., Pittsburg.

*Demonstrations.*—" Pivoting all Porcelain Crowns," by N. G. A. Bonwill, M. D., Philadelphia. "Pivoting Weston's Crowns." " The Application of Gold and Amalgam in Combination," by H. C. Register, M. D., Philadelphia.

Baltimoreans can secure excursion tickets by presenting orders at the following Stations, Union, Calvert, and Corner Baltimore and Calvert Sts.

W. H. FUNDENBERG,
Pittsburg, Pa. *Corresponding Sec'y*

---

### *Pittsburg Dental Association.*

The 8th, Annual Meeting of the Pittsburg Dental Association was held May 9th, 1882, at the office of Dr. Fundenburg, 323 Penn Ave. President Dr. W. F. Fundenberg presiding. By amendment the time of meeting was changed from 2nd, Tuesday to 2nd Thursday of each month. The following officers and delegates were elected for the ensuing year;

*President.* Dr. H. Manchester.

*Vice President,* Dr. J. Goshom.

*Secretary,* Dr. W. H. Fundenberg.

*Treasurer,* Dr. C. E. Diehl.

*Delegates to the American Dental Association,* Drs. F. A. Reinhart, F. Deterding, A. H. Greenawault, D. C. Phillips, and N. H. Fundenberg.

*Delegates to Pennsylvania State Dental Society,* Drs. J. Goshom, C. W. Beacom, F. A. Reinhart, Reineka and F. Troth.

Adjourned to meet 2nd, Thursday of September, 1882,

W. H. FUNDENBERG,
*Secretary.*

---

### *American Dental Association.*

The Twenty-second Annual Session of the American Dental Association will be held at Cincinnati, commencing Tuesdsy August 1st, 1882.

GEO. H. CUSHING, *Rec. Sec.*

*National Dental Association of the United States of America.*

The next meeting of the National Dental Association of the United States of America will be held in Washington, D. C., in the lecture hall of the National Museum of the Smithsonian Institution on the 3rd, 4th and 5th of August, 1882.

The meetings of the Association in Washington are quadrennial and international. A cordial invitation is extended to all members of the Profession in this and other countries to be present at this meeting.

R. FINLEY HUNT. D. D. S., *Sec'y.*

*Southern Dental Association.*

The Fourteenth Annual Meeting of the Southern Dental Association will be held in the building of the Baltimore College of Dental Surgery, Corner of Eutaw and Franklin Streets, Baltimore, Md., commencing Tuesday, August 8th prox. A cordial welcome is extended to all dentists to attend.

E. S. CHISHOLM, *Pres.*
W. H. HOFFMAN, *Sec'y.*

### To Readers and Correspondents.

Communications intended for publication, and exchange journals and papers, should be addressed to the EDITOR OF THE AMERICAN JOURNAL OF DENTAL SCIENCE, 259 N. Eutaw St., Baltimore, Md. All letters relating to the business of the journal, or containing cash remittances, to be directed to Messrs. SNOWDEN & COWMAN. Articles for publication should be received by the editor at least three weeks previous to the first of the month on which the number in which it is designed for them to appear, is issued.

### CONTENTS:

ART. I.—Treatment of the Teeth with Dead or Dying Pulps, and Alveolar Abscess. By Dr. H. H. Townsend.........49

ART. II.—Origin, Definition and Division of Tissues. By Dr. C. Heitzman.........54

ART. III.—Operative Dentistry. By Geo. S. Mills, D. D. S.........70

ART. IV.—What is Facial Neuralgia? By Parsons Shaw, D. D. S.........78

ART. V.—Removal of Naso-Pharyngeal Polypus. By Dr R. F. Weir.........83

Pennsylvania State Dental Society.........86

Pittsburg Dental Association.........87

### MONTHLY SUMMARY.

Dental Surgeons in the Army and Navy.........93
Therapeutic uses of Nitrite of Amyl.........94
A New Fungus.........95
Therapeutic Effects of Oxygen.........96
A Triumph of Dentistry.........96
College Examination for the Degree of Doctorate.........96

☞The American Journal of Dental Science will contain fifty pages of reading matter in each number, making a volume of about

*Six Hundred* pages,

EXCLUSIVE OF ADVERTISEMENTS.

THE

### AMERICAN JOURNAL

OF

### DENTAL SCIENCE.

The *Journal* is issued on the first of each month and contains not less than *fifty* pages of reading matter in each number, exclusive of advertisements, making a volume of *six hundred* pages per annum.

In each number will be found Original Communications, Correspondence, Selected Articles, Monthly Summary, Bibliographical Notices and Editorial Matter, and every effort will be exerted to render the *Journal* worthy of the patronage of the Dental profession.

A generous co-operation is respectfully solicited from the members of the profession; and as the *Journal* has now a printing office of its own, which materially lessens the cost of its publication, it will be issued at the reduced subscription price of

**$2.50 Per Annum in Advance.**

Communications are respectfully solicited on all subjects pertaining to the practice of dentistry, as well as reports of cases occurring in dental practice.

### RATES OF ADVERTISING.

| | 1 MO. | 2 MO. | 3 MO. | 6 MO. | 12 MO. |
|---|---|---|---|---|---|
| 1 PAGE. | $15 00 | $22 50 | $30 00 | $50 00 | $100 00 |
| ½ " | 10 00 | 15 00 | 20 00 | 30 00 | 50 00 |
| ¼ " | 7 00 | 11 00 | 15 00 | 20 00 | 30 00 |
| ⅛ " | 5 00 | 8 00 | 11 00 | 15 00 | 20 00 |

All original communications designed for publication, works for review, new instruments for notice, exchanges, &c., should be directed to the editor, 259 N. Eutaw St., Baltimore, Md.

All advertisements and subscriptions should be addressed to

SNOWDEN & COWMAN,

*Publishers of the Am. Journal of Dental Science.*

86 W. FAYETTE ST. BET. CHARLES & LIBERTY STS.,

BALTIMORE, MD.

